# Drug-resistant tuberculosis and pulmonary co-infections in immunocompromised patients: from multi-omics to precision therapy

**DOI:** 10.3389/fmicb.2026.1893416

**Published:** 2026-08-04

**Authors:** Ahong Wang, Hanizaier Reheman, Xiangtao Chen, Moru Shang, Dilinuer Abulikemu, Huaizhen Wang

**Affiliations:** 1 Department of Respiratory and Critical Care Medicine, Kashi Prefecture Second People’s Hospital, Kashi, Xinjiang, China; 2 Traditional Medicine Hospital of Kashgar Prefecture, Kashgar, China

**Keywords:** drug-resistant tuberculosis, immunocompromised patients, multi-omics, precision therapy, pulmonary co-infections

## Abstract

Drug-resistant tuberculosis remains a major global health threat, with an estimated 400,000 people developing rifampicin-resistant/multidrug-resistant tuberculosis (RR/MDR-TB) worldwide in 2023, according to the WHO Global Tuberculosis Report 2024. Immunocompromised populations, including people living with HIV, transplant recipients, patients receiving immunosuppressive therapies, and individuals with chronic metabolic diseases, are at particularly high risk of severe disease and pulmonary co-infections, resulting in delayed diagnosis, increased treatment complexity, and poor clinical outcomes. Despite advances in therapeutics, management remains constrained by fragmented diagnostic pathways, limited pathogen resolution, antimicrobial toxicity, and clinically significant drug–drug interactions. Recent progress in multi-omics technologies is reshaping understanding of host–pathogen dynamics in tuberculosis and co-infection states. Whole-genome sequencing enables rapid resistance prediction and transmission tracking, whereas transcriptomic, proteomic, metabolomic, and single-cell approaches are identifying biomarkers of disease severity, immune dysregulation, treatment response, and relapse risk. Parallel advances in metagenomic diagnostics and artificial intelligence-assisted imaging offer opportunities for earlier detection of mixed infections and improved clinical triage. Therapeutic paradigms are also evolving. Shorter all-oral regimens, individualized dosing strategies, therapeutic drug monitoring, and integrated antimicrobial stewardship are improving outcomes for resistant tuberculosis. Adjunctive approaches, including host-directed therapies, immunomodulation, inhaled drug delivery systems, and data-guided precision prescribing, may further enhance efficacy while reducing toxicity in vulnerable patients with co-infections. However, implementation remains uneven, and prospective evidence in immunocompromised populations is limited. Recent advances in multi-omics technologies including whole-genome sequencing, metagenomics, transcriptomics, proteomics, metabolomics, single-cell omics, and artificial intelligence-assisted diagnostics are transforming the diagnosis, biological stratification, and clinical management of DR-TB. In parallel, precision therapeutic approaches, including individualized regimen selection, therapeutic drug monitoring, host-directed therapies, and data-guided clinical decision-making, are enabling more personalized treatment strategies. This review integrates these advances into a precision medicine framework and discusses their clinical application, current limitations, and future directions for improving outcomes in immunocompromised patients with DR-TB and pulmonary co-infections.

## 1 Introduction

Tuberculosis (TB) remains the leading cause of death from a single infectious agent globally, with an estimated 10.7–10.8 million cases and approximately 1.25 million deaths in 2023, while multidrug-resistant and rifampicin-resistant TB continues to pose a major public health threat, causing approximately 150,000 deaths annually (Dean et al., 2022; Goletti et al., 2025). The persistence of transmission, delayed case detection, health-system disruption, and the expanding burden of antimicrobial resistance continue to impede progress toward global tuberculosis control targets (Dheda et al., 2017). Of particular concern is the ongoing rise of DR-TB, which encompasses rifampicin-resistant tuberculosis (RR-TB), defined as tuberculosis caused by *Mycobacterium tuberculosis* (*M. tuberculosis*) resistant to rifampicin with or without resistance to other first-line drugs; MDR-TB, defined as resistance to at least both rifampicin and isoniazid; pre-extensively drug-resistant tuberculosis (pre-XDR-TB), defined as MDR/RR-TB with additional resistance to any fluoroquinolone; and extensively drug-resistant tuberculosis (XDR-TB), defined as MDR/RR-TB with additional resistance to any fluoroquinolone and at least one additional Group A drug (bedaquiline or linezolid), according to the updated WHO definitions. Collectively, these forms represent one of the most formidable challenges in contemporary antimicrobial resistance (Dean et al., 2022; The Lancet Infectious Diseases, 2025). In 2023, an estimated 400,000 (95% UI: 360,000–440,000) individuals developed rifampicin-resistant/multidrug-resistant tuberculosis globally, with treatment success rates remaining substantially lower than for drug-susceptible disease (Antimicrobial Resistance Collaborators, 2022; Estaji et al., 2025). Compared with drug-susceptible disease, DR-TB is associated with longer treatment duration (often ≥ 18 months in conventional regimens), greater toxicity, higher costs, lower treatment success rates (approximately 60% globally), and increased mortality (Ahmad Mughal et al., 2025). The burden of severe disease is not evenly distributed, with the highest incidence and mortality observed in low- and middle-income countries and among vulnerable populations (Abdi et al., 2025). Immunocompromised populations experience disproportionate morbidity and mortality from TB because protective host responses required for containment of *M. tuberculosis* are weakened or dysregulated (Pai et al., 2016). These populations include people living with HIV, recipients of solid-organ or hematopoietic stem-cell transplants, patients receiving corticosteroids, biologics, chemotherapy, or other immunosuppressive agents, and individuals with diabetes, chronic kidney disease, cirrhosis, malnutrition, or advanced age (Pai et al., 2016; Dheda and Lange, 2024). In such hosts, defects in macrophage activation, impaired antigen presentation, T-cell dysfunction, altered cytokine signaling, and barrier injury increase susceptibility to primary infection, reactivation, disseminated disease, and poor microbiological clearance (O’Garra et al., 2013; Pai et al., 2016). An additional and increasingly recognized challenge is the high frequency of pulmonary co-infections in immunocompromised patients with tuberculosis (World Health Organization [WHO], 2024). Structural lung damage, impaired mucociliary clearance, prolonged hospital exposure, repeated antimicrobial use, and immune dysregulation create conditions that favor secondary or concurrent infection with bacterial, fungal, and viral pathogens (Hagman et al., 2025; Zhu et al., 2025). Common bacterial co-infections include *Pseudomonas aeruginosa*, *Staphylococcus aureus*, *Klebsiella pneumoniae*, and *Acinetobacter baumannii*, particularly in patients with prior hospitalization or chronic lung disease (Lansbury et al., 2020). Fungal pathogens such as *Aspergillus* spp., *Candida* spp., Mucorales, and *Cryptococcus* spp. are especially relevant in advanced immunosuppression, whereas respiratory viruses including influenza, SARS-CoV-2, and cytomegalovirus can precipitate rapid clinical deterioration (Drain et al., 2018). These overlapping infections often present with non-specific symptoms and radiological findings that mimic tuberculosis progression, leading to diagnostic delay and inappropriate empiric therapy (Dheda et al., 2017). Conventional diagnostic pathways are frequently inadequate for this complexity (Walzl et al., 2018). Sputum microscopy has limited sensitivity in paucibacillary disease, culture remains slow, and targeted molecular assays identify only a subset of resistance determinants or co-pathogens (Wobudeya et al., 2022). In immunocompromised patients, low pathogen burden, extrapulmonary involvement, prior antimicrobial exposure, and difficulty obtaining respiratory samples further reduce diagnostic yield. Consequently, clinicians must often make high-stakes therapeutic decisions with incomplete microbiological information while balancing toxicity, drug–drug interactions, and competing comorbidities (Meng et al., 2025). Recent advances in multi-omics and computational medicine offer a route beyond these limitations (Miotto et al., 2017). Whole-genome sequencing enables rapid identification of resistance mutations and transmission networks; transcriptomic and single-cell approaches characterize immune dysfunction and predict treatment response; proteomics and metabolomics are enabling biomarker discovery for disease severity and relapse; and metagenomic sequencing allows unbiased detection of mixed infections. Parallel developments in artificial intelligence-assisted imaging, clinical decision support systems, and therapeutic drug monitoring are beginning to support more individualized and precise care pathways (Saxena et al., 2025).

This Review examines the evolving landscape of drug-resistant tuberculosis and pulmonary co-infections in immunocompromised patients, with emphasis on evidence published in recent years. We synthesize advances in epidemiology, immunopathogenesis, diagnostics, and therapeutics, and discuss how precision medicine strategies might be implemented across diverse health-care settings. We also identify key evidence gaps, including the under-representation of immunocompromised populations in clinical trials, the scarcity of integrated studies of co-infection, and the need for scalable innovations applicable to resource-limited environments. The remainder of this review is organized as follows. Section 2 summarizes the global epidemiology and burden of drug-resistant tuberculosis in immunocompromised populations. Section 3 discusses the immunopathogenesis underlying increased susceptibility to tuberculosis and pulmonary co-infections. Section 4 reviews the epidemiology, clinical significance, and host-specific patterns of pulmonary co-infections. Section 5 highlights recent advances in diagnostic technologies, with emphasis on multi-omics approaches and artificial intelligence-assisted diagnostics. Section 6 focuses on precision therapeutic strategies, including optimized anti-tuberculosis regimens, therapeutic drug monitoring, management of co-infections, and host-directed therapies. Section 7 discusses disease management in major immunocompromised populations, and Section 8 outlines current research gaps and future directions for implementing precision medicine in drug-resistant tuberculosis.

### 1.1 Literature search strategy

This narrative review was developed through a comprehensive search of the published literature to summarize recent advances in DR-TB, pulmonary co-infections, immunopathogenesis, multi-omics technologies, and precision medicine in immunocompromised populations. Literature searches were conducted using PubMed/MEDLINE, Web of Science, Scopus, and Google Scholar. Priority was given to studies published between January 2015 and December 2025, while seminal earlier publications were included when essential to provide historical context or describe landmark discoveries. Search terms included combinations of *drug-resistant tuberculosis*, *multidrug-resistant tuberculosis*, *rifampicin-resistant tuberculosis*, *pulmonary co-infection*, *immunocompromised host*, *HIV*, *transplantation*, *diabetes mellitus*, *multi-omics*, *whole-genome sequencing*, *metagenomic next-generation sequencing*, *transcriptomics*, *proteomics*, *metabolomics*, *single-cell sequencing*, *host-directed therapy*, and *precision medicine*. Priority was given to recent systematic reviews, meta-analyses, clinical trials, international guidelines, and high-quality original research published in peer-reviewed journals. Relevant publications from the World Health Organization (WHO) and other authoritative organizations were also included where appropriate.

## 2 Epidemiology and global burden

**FIGURE 1:**
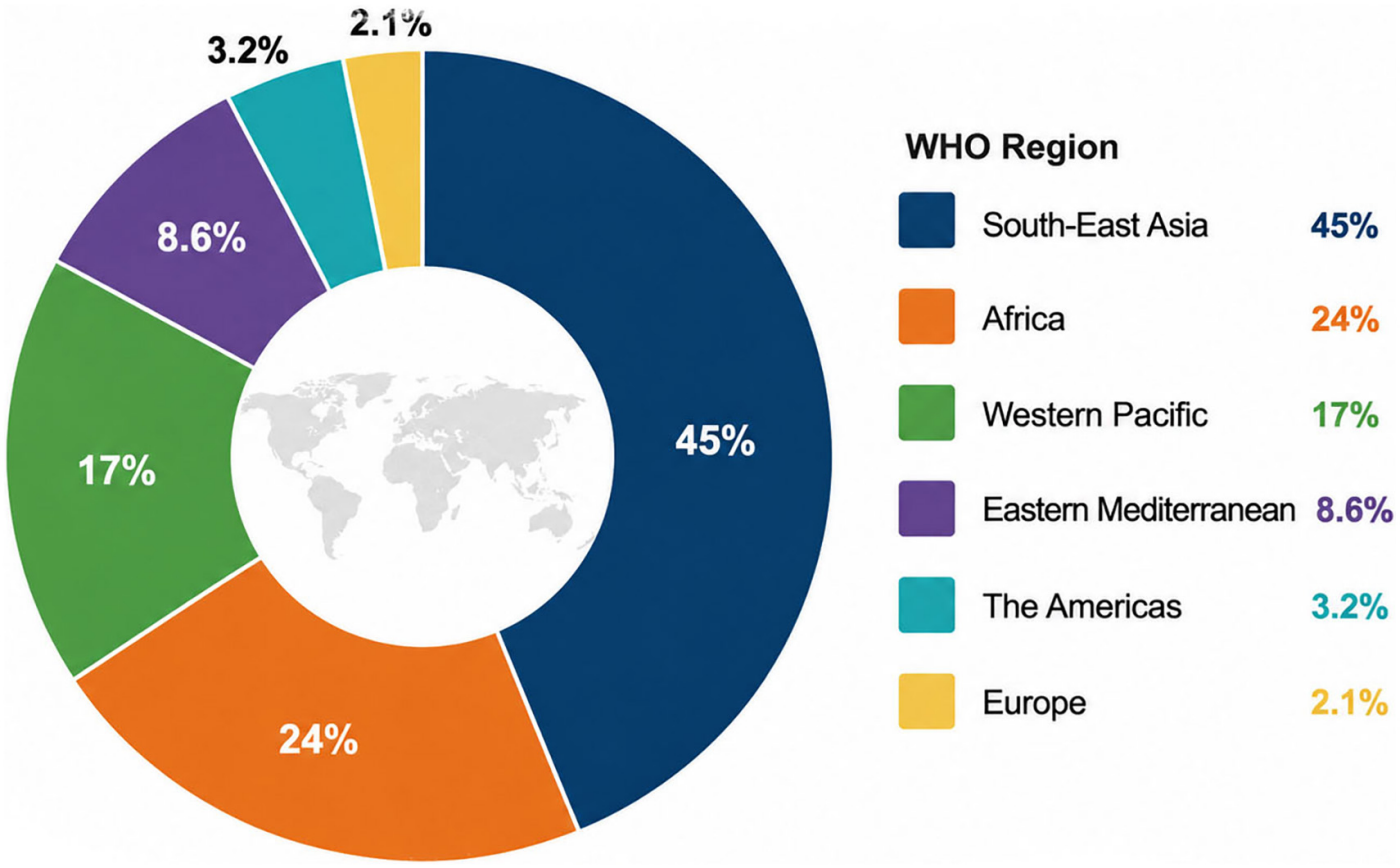
Global epidemiology of tuberculosis and drug-resistant tuberculosis in immunocompromised populations. Distribution of global incident tuberculosis cases across WHO regions in 2023. Percentages are based on WHO regional estimates of incident tuberculosis cases reported in the World Health Organization [WHO], 2025 WHO Global Tuberculosis Report 2024.

Drug-resistant tuberculosis remains a leading cause of infectious disease mortality and a major obstacle to tuberculosis elimination strategies. In 2023, an estimated 10.8 million people developed tuberculosis worldwide and approximately 1.25 million deaths were attributed to the disease (Wei and Zhang, 2024; Suvvari, 2025). Of these, about 400,000 individuals developed RR-TB/MDR-TB, highlighting the continuing expansion of antimicrobial resistance within tuberculosis control programs (Vishwakarma et al., 2023). Although expanded molecular testing and decentralized diagnostic platforms have improved case detection in several high-burden countries, including India, China, Indonesia, the Philippines, Pakistan, the Russian Federation, and South Africa, major gaps persist between estimated disease burden and treatment enrollment (Figure 1; Goletti et al., 2025). In 2023, only a fraction of the people estimated to have MDR/RR-TB were diagnosed and initiated on appropriate therapy; globally, 175,923 individuals were enrolled on treatment, representing a slight decrease from 177,912 in 2022. This persistent treatment gap reflects ongoing barriers related to health-system fragility, conflict, supply-chain disruption, limited laboratory capacity, and incomplete post-pandemic service recovery (Matteelli et al., 2025). This diagnostic and treatment gap is especially consequential in immunocompromised populations, in whom delayed recognition can rapidly translate into severe disease, dissemination, and death. Geographical heterogeneity remains pronounced (Figure 1). South-East Asia accounted for approximately 45% of incident tuberculosis cases globally in 2023, while the WHO African Region accounted for about 24% and the Western Pacific Region for 17%, highlighting the concentration of burden in South and Southeast Asia (Bajpai et al., 2025). This regional distribution reflects large population size, dense urban transmission, and the increasing prevalence of diabetes and other chronic diseases (Kumar et al., 2024; Goletti et al., 2025). Sub-Saharan Africa continues to experience a high burden of tuberculosis closely linked to HIV co-infection, accounting for an estimated 23–24% of global incident, while the WHO African Region represented approximately 73% of all tuberculosis cases occurring among people living with HIV. In these settings, immune suppression increases the likelihood of atypical presentation, smear-negative disease, disseminated infection, and early mortality (Telisinghe et al., 2016; Kapata et al., 2025). Eastern Europe and parts of central Asia remain important epicenters of rifampicin-resistance, shaped by historical treatment interruption, incarceration-associated transmission, and variable access to newer therapeutics (Balinska, 2000). In Latin America and the Middle East, burden patterns are more heterogeneous but are increasingly influenced by migration, socioeconomic instability, and unequal diagnostic access (Rotundo et al., 2026). The epidemiology of DR-TB is closely linked to the expanding global population living with impaired immunity. HIV remains the most established risk factor for severe and recurrent tuberculosis, particularly when diagnosis is delayed or antiretroviral therapy is interrupted. However, the profile of vulnerability has broadened substantially. Increasing use of biologic and targeted immunosuppressive therapies including tumor necrosis factor inhibitors (infliximab, adalimumab, and etanercept), interleukin-6 receptor antagonists (tocilizumab), anti-CD20 monoclonal antibodies (rituximab), and Janus kinase inhibitors (tofacitinib, baricitinib, and ruxolitinib) together with transplantation programs requiring calcineurin inhibitors (tacrolimus and ciclosporin) and intensive anticancer regimens such as cytotoxic chemotherapy or CAR T-cell therapy, has created new patient groups at risk of tuberculosis reactivation or *de novo* infection (Robert and Miossec, 2021; Ji et al., 2022; Alıravcı et al., 2025). In parallel, the worldwide rise in diabetes, chronic kidney disease, chronic lung disease, malnutrition, and population aging has enlarged the pool of individuals with functional immune impairment. These shifts indicate that future tuberculosis control strategies must move beyond traditional risk categories and incorporate a wider framework of immune vulnerability (Zou et al., 2025). Pulmonary co-infections add an additional layer of epidemiological complexity (Table 1). Post-COVID-19 bacterial superinfection has been repeatedly associated with structural lung damage (including cavitary change and bronchiectasis), prolonged hospitalization, prior broad-spectrum antibiotic exposure, intensive-care admission, mechanical ventilation, and other forms of invasive respiratory support. Gram-negative pathogens such as *Klebsiella pneumoniae*, *Pseudomonas aeruginosa*, and *Acinetobacter baumannii* are frequently reported in tertiary-care settings, whereas *Staphylococcus aureus* remains an important cause of secondary infection in post-viral states and chronic lung disease (Chong et al., 2021; Grasselli et al., 2021; Patil et al., 2023). Fungal disease is increasingly recognized but likely underdiagnosed. Chronic pulmonary aspergillosis can complicate residual cavities after tuberculosis, whereas invasive aspergillosis, mucormycosis, and candidiasis are concentrated in transplant recipients, patients with hematological malignancy, prolonged corticosteroid exposure, or uncontrolled diabetes (Klinting et al., 2022). Viral co-infections, particularly influenza, SARS-CoV-2, and cytomegalovirus in selected hosts, have also been associated with excess respiratory failure and interruption of TB care pathways (Dasan et al., 2025; Quintana-Ospina et al., 2025). Outcomes in immunocompromised patients with DR-TB and co-infections are consistently worse than in immunocompetent populations. Mortality is driven by delayed diagnosis, severe baseline comorbidity, sepsis, respiratory insufficiency, and inability to tolerate prolonged multidrug regimens (Vonineng et al., 2025). Hospital stays are longer, recurrence rates are higher, and treatment completion is less likely when patients face competing toxicities, pill burden, clinically significant drug–drug interactions. In resource-limited settings, these challenges are magnified by restricted access to bronchoscopy, fungal diagnostics, therapeutic drug monitoring, and intensive care (Pooranagangadevi and Padmapriyadarsini, 2022; Ryu et al., 2024; Rashid et al., 2021). Expanded use of rapid molecular tests, shorter all-oral regimens, decentralized treatment models, and integration of TB services with HIV and chronic disease programs have improved outcomes in several settings. Yet epidemiological data remain fragmented. Many surveillance systems do not systematically capture immune status, co-infecting pathogens, antifungal resistance, treatment toxicity, or long-term outcomes. Standardized prospective registries integrating microbiology, host factors, and longitudinal outcomes are urgently needed. Without better data, the true burden of DR-TB and pulmonary co-infections in immunocompromised populations will remain underestimated, and opportunities for targeted prevention and precision care will continue to be missed.

**TABLE 1:** Recent epidemiological studies.

Year	Country/ Region	Population	Co-infection/ Condition	Verified key findings	References
2026	India	Pulmonary TB patients evaluated for fungal disease	Pulmonary mycotic co-infection	Fungal co-infection was significantly higher in MDR-TB patients than drug-susceptible TB patients (60.0% vs. 42.6%). Immunocompromised patients had higher fungal infection rates than immunocompetent patients (70.4% vs. 39.5%).	(Jigan et al., 2026)
2025	Global	GBD 2021 MDR/XDR-TB analysis	MDR-TB and XDR-TB burden	Global burden of MDR-TB and XDR-TB remained disproportionately high in low- and lower-middle SDI regions, with substantial regional inequities.	(Tan et al., 2025)
2022	Global	WHO TB surveillance data	Drug-resistant TB	WHO estimated that MDR/RR-TB continues to represent a major global health burden despite gradual declines in proportion among new and previously treated TB cases.	(Dean et al., 2022)
2024	Global	GBD 2019 dataset	MDR-TB epidemiology	Global MDR-TB prevalence and mortality burden increased substantially between 1990 and 2019, especially in Sub-Saharan Africa and low-SDI regions.	(Lv et al., 2024)
2025	Global	Smoking-associated MDR-TB burden	MDR-TB with risk factors	Smoking-attributable MDR-TB burden remained highest in low and low-middle SDI regions.	(Dan et al., 2025)
2025	China	National TB burden modeling	HIV-MDR-TB and HIV-XDR-TB	Forecast modeling projected continued increases in HIV-MDR-TB and HIV-XDR-TB burden through 2035.	(Zhang et al., 2025)
2025	Global	Children and adolescents	MDR-TB burden	Global MDR-TB burden among children and adolescents increased from 1990 to 2019, especially in lower SDI regions.	(Zhong et al., 2025)

SDI, Socio-demographic Index; GBD, Global Burden of Disease; MDR-TB, multidrug-resistant tuberculosis; XDR-TB, extensively drug-resistant tuberculosis.

## 3 Immunopathogenesis: why the immunocompromised lung fails

**FIGURE 2:**
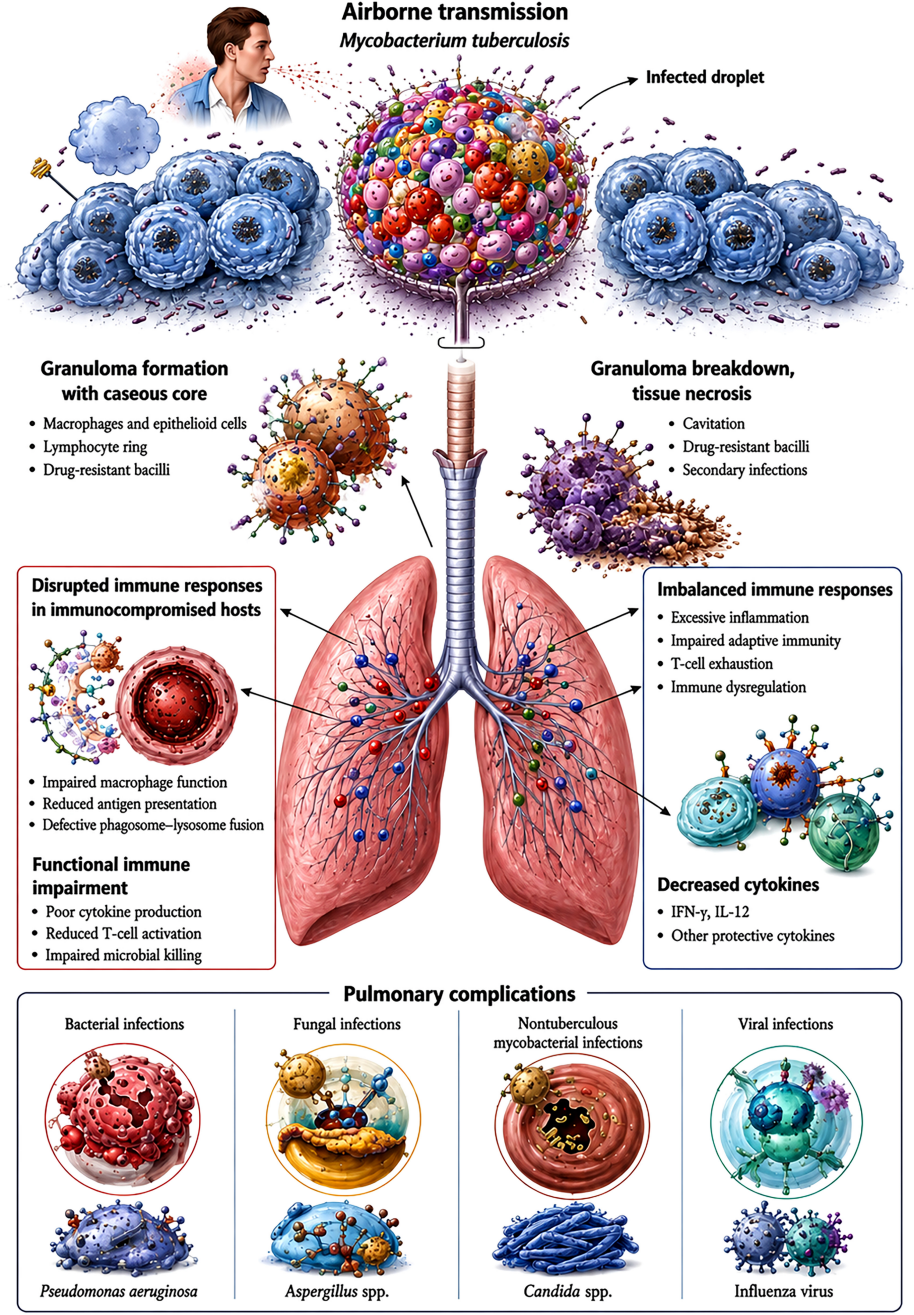
Mechanistic model of host immune dysfunction enabling drug-resistant tuberculosis and pulmonary co-infections in immunocompromised patients. Conceptual illustration depicting the pathophysiological mechanisms by which impaired host immunity promotes the progression of DR-TB and increases susceptibility to pulmonary co-infections. Following inhalation of *M. tuberculosis*, impaired macrophage function, defective antigen presentation, reduced T-cell activation, and dysregulated cytokine production compromise granuloma integrity, resulting in tissue necrosis, cavitation, and enhanced bacterial persistence. These immune defects increase vulnerability to secondary bacterial, fungal, viral, and NTM infections, leading to further pulmonary damage and disease progression. Arrows indicate the sequential progression from infection to immune dysfunction and pulmonary complications. DR-TB, drug-resistant tuberculosis; IFN-γ, interferon-gamma; IL, interleukin; NTM, non-tuberculous mycobacteria. Figure created using GPT-AI and manually refined by the authors. All scientific content, pathways, and workflows were verified against the peer-reviewed literature cited in the manuscript.

Protection against tuberculosis depends on co-ordinated innate and adaptive immune responses that contain *M. tuberculosis* within organized granulomatous lesions while limiting collateral lung injury. In immunocompromised patients, this balance is disrupted by quantitative or qualitative defects in host defense, permitting uncontrolled bacillary replication, impaired granuloma integrity, dissemination, and heightened susceptibility to secondary pathogens. Rather than a single pathway, failure of the immunocompromised lung reflects convergent dysfunction across macrophages, dendritic cells, T lymphocytes, neutrophils, epithelial barriers, and the local microbiome (O’Garra et al., 2013; Pai et al., 2016; Figure 2).

### 3.1 Cellular immune failure and pathogen persistence

Alveolar macrophages are the first major cellular niche encountered after inhalation of *M. tuberculosis*. Under effective immunity, phagocytosis, phagolysosomal maturation, autophagy, nitric oxide signaling, and cytokine cross-talk restrict intracellular survival (Imran et al., 2025). Recent transcriptomic and single-cell studies indicate that macrophages in active tuberculosis exhibit a dysregulated phenotype characterized by persistent inflammatory activation together with impaired antimicrobial and antigen-presenting functions. These abnormalities are further amplified in immunocompromised hosts, contributing to inadequate bacterial clearance, granuloma instability, and increased susceptibility to secondary pulmonary infections. This mixed phenotype suggests a state of inflammatory activation that contributes to tissue injury but remains permissive to incomplete bacterial clearance (Gideon et al., 2015; Moreira-Teixeira et al., 2018). Dendritic-cell dysfunction further compromises protective immunity in immunocompromised patients. Reduced antigen presentation and impaired T-cell priming, particularly in advanced HIV infection, transplantation, hematological malignancy, and during treatment with TNF inhibitors or Janus kinase inhibitors, weaken granuloma maintenance and increase susceptibility to tuberculosis reactivation, disseminated disease, and pulmonary co-infections (O’Garra et al., 2013; Getahun et al., 2015; Pai et al., 2016). CD4-positive T cells are central to control of *M. tuberculosis* through production of interferon-γ, tumor necrosis factor, and interleukin-2, which activate infected macrophages and sustain granuloma structure. CD8-positive T cells contribute through perforin- and granzyme-mediated killing of infected host cells and by secretion of interferon-γ. Quantitative loss of these lymphocyte populations, as seen in advanced HIV infection, lymphodepleting chemotherapy, or post-transplant immunosuppression, is strongly associated with disseminated disease, higher bacillary burden, and increased mortality. Persistent mycobacterial antigen exposure can additionally induce T-cell exhaustion, characterized by reduced production of interferon-γ and tumor necrosis factor, impaired proliferative capacity, metabolic dysfunction, and increased expression of inhibitory receptors including PD-1, TIGIT, CTLA-4, and LAG-3. These dysfunctional immune states may compromise bacterial clearance, delay culture conversion, and increase the risk of treatment failure or post-treatment relapse despite microbiologically active regimens (Wherry and Kurachi, 2015; Halliday et al., 2021; Figure 2). The recognition of T-cell exhaustion also has important therapeutic implications. Restoration of T-cell function through carefully targeted host-directed therapies, including modulation of immune checkpoint pathways, represents an emerging strategy to enhance antimycobacterial immunity while limiting excessive inflammation. Although immune checkpoint modulation has shown encouraging results in preclinical tuberculosis models, clinical evidence remains limited, and concerns regarding immune-mediated pathology indicate that these approaches should currently be considered investigational until supported by adequately designed clinical trials (Wherry and Kurachi, 2015; Cubillos-Angulo et al., 2022). Emerging evidence also indicates that trained immunity contributes to the host response against *M. tuberculosis*. Unlike classical adaptive immune memory, trained immunity involves long-term functional reprograming of innate immune cells, particularly monocytes, macrophages, and natural killer cells, through epigenetic and metabolic remodeling following exposure to microbial stimuli such as Bacille Calmette–Guérin (BCG) vaccination or *M. tuberculosis* (Kaufmann et al., 2018; Netea et al., 2020). This enhanced innate responsiveness can improve antimicrobial activity and cytokine production upon secondary challenge; however, excessive or dysregulated trained immune responses may also amplify inflammatory tissue damage and influence disease progression. Understanding the balance between protective and pathological trained immunity may facilitate the development of novel host-directed therapeutic strategies and improve precision medicine approaches for immunocompromised patients with drug-resistant tuberculosis (Moorlag et al., 2019).

### 3.2 Tissue injury, barrier disruption, and co-infection susceptibility

Although neutrophils contribute to early host defense against *M. tuberculosis*, excessive or sustained neutrophilic inflammation is increasingly recognized as a major driver of lung injury in severe tuberculosis. Blood and airway neutrophil signatures correlate with cavitary disease, high bacillary burden, and poor treatment outcomes. Mechanistically, release of matrix metalloproteinases (particularly MMP-8 and MMP-9), neutrophil elastase, myeloperoxidase, and neutrophil extracellular traps can degrade extracellular matrix, amplify necrotizing inflammation, and impair alveolar capillary gas exchange. In immunocompromised patients with bacterial superinfection, tuberculosis-associated tissue destruction may facilitate secondary bacterial adherence and growth, while concurrent sepsis further intensifies inflammatory injury and respiratory failure (Lowe et al., 2012; Ong et al., 2014). The healthy respiratory tract depends on intact epithelial barriers, coordinated mucociliary clearance, surfactant proteins, antimicrobial peptides, and a balanced airway microbiome. Prior tuberculosis, tobacco smoke exposure, diabetes, malnutrition, HIV, and repeated antibiotic exposure can disrupt each of these protective systems. Post-tuberculosis structural lung disease including residual cavities, fibrosis, bronchiectasis, and airway distortion creates persistent ecological niches for opportunistic pathogens such as *Aspergillus fumigatus*, *Pseudomonas aeruginosa*, non-tuberculous mycobacteria, and other healthcare-associated organisms. Chronic pulmonary aspergillosis is a particularly important complication of residual cavities after tuberculosis, whereas invasive mold disease is concentrated in profoundly immunosuppressed hosts (Ortiz-Brizuela and Ponce-de-León, 2018; Lipinksi et al., 2024; Al-Hindawi et al., 2026). Emerging microbiome and multi-omics studies further indicate that tuberculosis is associated with loss of microbial diversity and enrichment of inflammatory pathobionts within the airway. Parallel disruption of the gut–lung axis may alter short-chain fatty acid signaling, systemic immune tone, and mucosal defense, thereby increasing susceptibility to secondary infection and prolonging inflammatory recovery after microbiological cure. These findings support an ecological model in which tuberculosis is both a pathogen-specific disease and a destabilizer of respiratory ecosystem resilience (Dang and Marsland, 2019). The dominant pathway of tissue failure differs across host phenotypes (Table 2). The mechanisms of immune dysfunction differ across immunocompromised populations and influence susceptibility to bacterial, fungal, and viral pulmonary co-infections. These host-specific differences have important implications for diagnosis, treatment, and prognosis and are discussed in greater detail in section 7 (Getahun et al., 2015; Pai et al., 2016).

**TABLE 2:** Common pulmonary co-infections in immunocompromised patients with drug-resistant tuberculosis: pathogens, diagnostic clues, resistance concerns, and treatment considerations .

Pathogen	Major species/syndrome	High-risk host groups	Diagnostic clues	Resistance concerns	Preferred treatment/notes	References
Bacterial	*Klebsiella pneumoniae* pneumonia	ICU patients, diabetes, prolonged admission, prior antibiotics	New lobar consolidation, sepsis, purulent sputum, rapid deterioration	ESBL and carbapenem resistance increasingly reported globally	Culture-guided beta-lactam therapy; carbapenem-sparing when possible; source control	(Wyres and Holt, 2018)
Bacterial	*Pseudomonas aeruginosa* lower respiratory infection	Bronchiectasis, prior TB cavities, ventilation, chronic lung disease	Increased sputum, recurrent exacerbations, cavitary superinfection	Multidrug resistance common after repeated antibiotic exposure	Antipseudomonal therapy guided by susceptibility; inhaled adjuncts in selected chronic disease	(Bassetti et al., 2018)
Bacterial	*Acinetobacter baumannii* hospital-acquired pneumonia	ICU, ventilated, transplant, prolonged hospital stay	Ventilator-associated pneumonia, severe hypoxemia, sepsis	Extensive drug resistance common in tertiary-care settings	Combination therapy based on susceptibility and local epidemiology	(Tacconelli et al., 2018)
Bacterial	*Staphylococcus aureus* (including MRSA)	Post-viral infection, hemodialysis, device-related care	Necrotizing pneumonia, empyema, rapid inflammatory progression	MRSA prevalence varies by region	Anti-staphylococcal therapy; add MRSA coverage when clinically indicated	(Tong et al., 2015)
Fungal	Chronic pulmonary aspergillosis	Prior TB cavities, diabetes, chronic lung disease	Hemoptysis, weight loss, enlarging cavity, fungal ball	Azole resistance increasingly recognized in some regions	Oral triazole therapy, surgery/embolization in selected hemoptysis	(Denning et al., 2016)
Fungal	Invasive aspergillosis	Transplant, hematological malignancy, corticosteroids	Halo sign, nodules, fever, hypoxia, positive galactomannan	Azole-resistant isolates reported; breakthrough infection possible	Voriconazole/isavuconazole guided by interactions and drug monitoring	(Patterson et al., 2016)
Fungal	Candidiasis (true invasive disease)	Neutropenia, ICU, central lines, broad antibiotics	Candidemia with pulmonary involvement uncommon; respiratory isolation often colonization	Species-dependent azole/echinocandin resistance	Treat only proven/probable invasive disease; avoid overtreatment of colonization	(Pappas et al., 2016)
Fungal	Mucormycosis	Uncontrolled diabetes, steroid exposure, transplant	Rapid tissue invasion, necrosis, sinus or pulmonary cavitation	Intrinsic resistance to many azoles	Liposomal amphotericin B plus urgent debridement when feasible	(Cornely et al., 2019)
Fungal	Cryptococcosis	Advanced HIV, transplant	Diffuse infiltrates, nodules, CNS symptoms may coexist	Fluconazole resistance uncommon but reported	Amphotericin-based induction or high-dose azole depending on severity	(Perfect et al., 2010)
Viral	SARS-CoV-2 co-infection	All groups, especially chronic disease/immunosuppression	Acute hypoxemia, diffuse infiltrates, thrombo-inflammatory state	Variant-dependent antiviral susceptibility	Early antiviral therapy when eligible; monitor TB treatment interruption	(Wu et al., 2023)
Viral	Influenza	Seasonal risk; elderly; chronic lung disease	Fever, myalgia, secondary bacterial pneumonia	Neuraminidase resistance uncommon but possible	Early oseltamivir or equivalent; vaccination prevention	(Uyeki et al., 2019)
Viral	Cytomegalovirus pneumonitis	Transplant, profound immunosuppression	Fever, diffuse ground-glass opacities, cytopenias	Antiviral toxicity and resistance in prolonged exposure	Ganciclovir/valganciclovir with virological monitoring	(Kotton et al., 2018)

ESBL, extended-spectrum beta-lactamase; ICU, intensive care unit; MRSA, meticillin-resistant *Staphylococcus aureus*; CNS, central nervous system.

### 3.3 Translational implications

These mechanisms have direct therapeutic relevance. Host-directed strategies targeting excessive neutrophilic inflammation, matrix metalloproteinase activity, epithelial repair, or dysfunctional macrophage responses could complement antimicrobial therapy. Biomarkers derived from transcriptomics, proteomics, metabolomics, and cellular phenotyping may enable stratification of patients according to dominant immune and tissue-injury endotypes rather than broad diagnostic labels alone. Combined with rapid microbiological diagnostics and therapeutic drug monitoring, such approaches are central to future precision medicine strategies for drug-resistant tuberculosis and pulmonary co-infections (Gilmour and Alene, 2024; Rie et al., 2026).

### 3.4 Bacterial, fungal, and viral co-infections: epidemiology, clinical burden, and management implications

Pulmonary co-infections are increasingly recognized as major contributors to adverse outcomes in patients with DR-TB, particularly among immunocompromised hosts. However, the true burden remains underestimated because many studies are retrospective, diagnostic testing is incomplete, and fungal or viral pathogens are not systematically sought. Available data nevertheless indicate that co-infection is clinically important across bacterial, fungal, and viral domains (Oliveira et al., 2025). Bacterial co-infection is the most frequently reported category in hospital-based cohorts. Across studies of severe pulmonary tuberculosis, post-tuberculosis lung disease, and intensive-care admissions, bacterial superinfection has been reported in approximately 15–35% of patients, with higher rates in mechanically ventilated populations and those with structural lung disease (Stewart and Kotton, 2024; Giannella et al., 2025). Gram-negative pathogens, *Klebsiella pneumoniae*, *Pseudomonas aeruginosa*, *Acinetobacter baumannii*, and *Escherichia coli* are predominated in tertiary-care settings, reflecting prior antibiotic exposure, healthcare contact, and device-associated risk (Kerimoglu et al., 2025). Methicillin resistant *Staphylococcus aureus*, remains important in post-viral states and recurrent hospital admissions. Secondary bacterial infection is associated with increased inflammatory injury, longer hospital stay, greater use of broad-spectrum antibiotics, and higher risk of sepsis and respiratory failure (Lucian-Daniel et al., 2026). Fungal co-infection is less frequently diagnosed but may be substantially under-recognized. Chronic pulmonary aspergillosis (CPA) is one of the most important long-term complications of prior pulmonary tuberculosis. A landmark global modeling study by Denning et al. (2011) estimated a 5-year global prevalence of approximately 1.17 million (range 0.85–1.37 million) cases of CPA following pulmonary tuberculosis. Subsequent studies have reported varying prevalence estimates depending on the modeling methodology, geographical setting, and diagnostic assumptions. Therefore, these values should be interpreted as model-based estimates rather than directly measured global prevalence (Denning et al., 2011). However, this estimate was derived using tuberculosis data and modeling assumptions from the late 2000s and should be interpreted as a historical reference rather than a current global burden estimate. More recent evidence indicates that CPA remains an important and frequently under-recognized complication of pulmonary tuberculosis, particularly in patients with residual post-tuberculosis lung disease. The true burden is likely underestimated because of limited access to fungal diagnostics, variability in case definitions, and heterogeneity in epidemiological study designs across different settings. Recent systematic reviews and expert commentaries have further emphasized the need for standardized diagnostic criteria, harmonized reporting methods, and improved surveillance to generate more reliable global burden estimates, especially in high tuberculosis-burden countries (Bongomin and Denning, 2025; Sehgal et al., 2025). In cohorts of patients with residual post-tuberculosis cavities, CPA prevalence commonly ranges from 10 to 20%, and may be higher in referral populations (Madden et al., 2025). Invasive aspergillosis is less common but carries high mortality in patients receiving prolonged corticosteroids, transplantation-related immunosuppression, or intensive chemotherapy. Mucormycosis should be considered in uncontrolled diabetes or profound immunosuppression, whereas *cryptococcosis* remains relevant in advanced HIV infection. Distinguishing airway colonization from invasive disease is especially important for *Candida* species, which are frequently cultured from respiratory samples but rarely cause primary pneumonia (Pourghasem et al., 2026). Respiratory viral co-infections can precipitate abrupt decompensation in DR-TB through increased airway inflammation, impaired gas exchange, and facilitation of secondary bacterial infection. During the COVID-19 pandemic, multiple international cohorts showed that tuberculosis–SARS-CoV-2 co-infection was associated with higher mortality than either disease alone, particularly in older adults and patients with HIV, diabetes, or chronic lung disease (Tadolini et al., 2020). Influenza remains an important seasonal trigger for hospitalization in vulnerable patients with chronic respiratory impairment. Cytomegalovirus pneumonitis should be considered in transplant recipients and profoundly immunosuppressed hosts presenting with diffuse infiltrates, fever, or unexplained hypoxemia (Stewart and Kotton, 2024). Diagnosis requires active suspicion because symptoms and imaging findings frequently overlap with progression of DR-TB. New consolidation, persistent fever, enlarging cavities, hemoptysis, unexplained hypoxemia, or failure to improve despite microbiologically active TB therapy should prompt reassessment for co-pathogens. Recommended investigations include repeat sputum culture, multiplex respiratory PCR, bronchoscopy with bronchoalveolar lavage in selected patients, fungal biomarkers (galactomannan, β-D-glucan), computed tomography, and blood cultures when sepsis is suspected (Aslam et al., 2020). Management depends on rapid pathogen identification, antimicrobial stewardship, and careful review of drug–drug interactions. In patients receiving rifamycins, bedaquiline, linezolid, azoles, calcineurin inhibitors, or antiretroviral therapy, pharmacokinetic interactions may be clinically significant and often require dose adjustment, substitution, or therapeutic drug monitoring. Prospective studies defining optimal integrated diagnostic pathways and precision treatment strategies for coinfected immunocompromised patients remain urgently needed (World Health Organization [WHO], 2024).

### 3.5 Host-specific co-infection patterns, clinical implications, and unmet needs

The spectrum of pulmonary co-infection differs substantially according to the underlying immune defect, and recognition of host phenotype is essential for rational diagnostic and therapeutic decision-making (Table 2). In people living with HIV, pulmonary tuberculosis frequently coexists with recurrent bacterial pneumonia, *Pneumocystis jirovecii* pneumonia (PJP), cryptococcosis, and other opportunistic fungal disease, particularly at low CD4 cell counts. WHO estimates indicate that the African Region accounts for the majority of tuberculosis cases among people living with HIV, emphasizing the importance of integrated TB–HIV diagnostic pathways (Pai et al., 2016). In patients with advanced HIV and diffuse infiltrates, failure to consider PJP, cytomegalovirus, or bacterial superinfection may delay life-saving therapy. In solid-organ and hematopoietic stem-cell transplant recipients, prolonged immunosuppression, calcineurin inhibitors, antimetabolites, corticosteroids, and graft-related immune dysfunction create a distinct respiratory risk profile. In these populations, invasive *aspergillosis*, *mucormycosis*, *cytomegalovirus pneumonitis, nocardiosis*, and nosocomial Gram-negative pneumonia are prominent differential diagnoses alongside tuberculosis (Patterson et al., 2016; Elhaj Mahmoud et al., 2024). Early bronchoscopy, fungal biomarkers, viral PCR, and therapeutic drug monitoring are often particularly valuable because symptoms may be subtle while progression can be rapid (Brown et al., 2025). In oncology populations, especially those receiving intensive chemotherapy, anti-CD20 therapy, stem-cell transplantation, or prolonged corticosteroids, neutropenia and lymphocyte dysfunction markedly increase susceptibility to mold disease, bacterial sepsis, and viral reactivation. Pulmonary infiltrates in this setting should not be attributed to tuberculosis alone without reassessment for invasive fungal infection or resistant bacterial pathogens (Brown et al., 2025; Rabagliati et al., 2025). In diabetes mellitus, chronic hyperglycemia impairs neutrophil chemotaxis, macrophage killing, and epithelial repair, thereby increasing susceptibility to tuberculosis and secondary infection. Chronic pulmonary aspergillosis and mucormycosis deserve particular attention, especially in patients with residual cavities, ketoacidosis, corticosteroid exposure, or poorly controlled glycemia. Diabetes is also associated with delayed sputum conversion, more extensive radiological disease, and worse tuberculosis outcomes (Martinez and Kornfeld, 2014). Older adults represent an additional high-risk group in whom immunosenescence, aspiration risk, frailty, chronic lung disease, and multimorbidity increase vulnerability to mixed infections and reduce tolerance of prolonged multidrug regimens. In this population, atypical presentations and polypharmacy further complicate diagnosis and management (Pai et al., 2016). These host-specific patterns should directly inform empirical testing strategies. Rather than applying a uniform diagnostic algorithm, clinicians should tailor investigations according to immune phenotype, exposure history, radiology, and local epidemiology. For example, diffuse bilateral infiltrates in advanced HIV should prompt evaluation for PJP and CMV; nodular or halo-sign lesions in transplant recipients should trigger urgent mold diagnostics; and new ventilator-associated consolidation in prolonged hospitalization should prompt assessment for multidrug-resistant Gram-negative pathogens. Management of pulmonary co-infections in DR-TB therefore requires a shift from single-pathogen thinking to integrated respiratory infectious disease care. Repeated microbiological reassessment, bronchoscopy in selected cases, antimicrobial susceptibility testing, antifungal stewardship, therapeutic drug monitoring, and multidisciplinary review are often necessary. However, prospective evidence remains scarce because many tuberculosis trials exclude severely immunocompromised patients or do not systematically capture non-mycobacterial pathogens. Future studies should evaluate combined diagnostic algorithms, pathogen-specific treatment thresholds, and outcomes of precision antimicrobial strategies in real-world DR-TB populations (Lange et al., 2020).

## 4 Diagnostic revolution: from conventional tests to multi-omics

### 4.1 Conventional and molecular diagnostics in DR-TB

**FIGURE 3:**
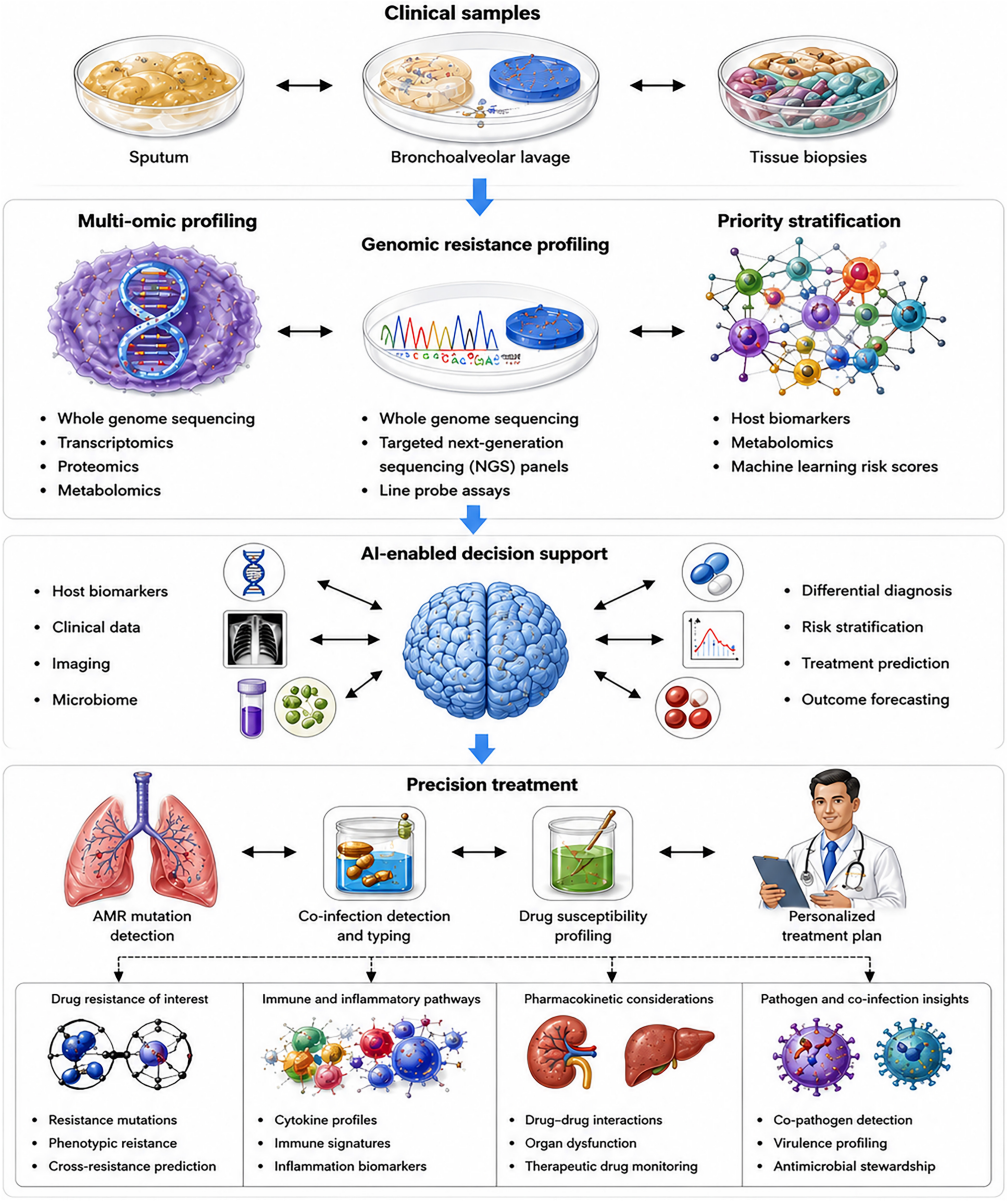
Integrated diagnostic pipeline for drug-resistant tuberculosis and pulmonary co-infections. Conceptual overview of a precision diagnostic workflow for immunocompromised patients with suspected DR-TB and pulmonary co-infections. Clinical specimens (sputum, bronchoalveolar lavage, and tissue biopsy) undergo integrated multi-omic profiling, genomic resistance testing, and biomarker analysis. Artificial intelligence (AI)-enabled clinical decision support combines molecular, microbiological, imaging, and clinical data to facilitate rapid diagnosis, resistance prediction, risk stratification, and individualized treatment planning. Arrows indicate the sequential diagnostic workflow and bidirectional integration of multi-source data into precision therapeutic decision-making. NGS, next-generation sequencing; AI, artificial intelligence; DR-TB, drug-resistant tuberculosis; AMR, antimicrobial resistance. Figure created using GPT-AI and manually refined by the authors. All scientific content, diagnostic pathways, and workflows were verified against the peer-reviewed literature cited in the manuscript.

Timely diagnosis of DR-TB with concurrent pulmonary co-infection in immunocompromised patients remains a defining unmet need in contemporary infectious diseases. In this population, diagnostic uncertainty is generated not by one missing assay but by the convergence of low bacillary burden, extrapulmonary dissemination, intermittent pathogen shedding, prior antimicrobial exposure, atypical imaging, and simultaneous infection with non-mycobacterial organisms. As a result, resistance is frequently recognized late, clinically important co-pathogens remain undetected, and treatment decisions are often made under conditions of incomplete microbiological certainty. The central shift in the field is therefore conceptual: from sequential pathogen testing towards integrated, data-rich characterization of pathogen, host, and disease state (Figure 3; World Health Organization [WHO], 2024).

Conventional tools remain necessary but are intrinsically constrained. Smear microscopy is scalable and inexpensive, yet performs poorly in paucibacillary disease, advanced HIV infection, and patients unable to produce representative sputum (Li et al., 2025). To facilitate translation of these advances into clinical practice, particularly in resource-constrained settings, a tiered implementation framework based on current WHO recommendations is proposed (Table 3). Mycobacterial culture remains the reference standard and is indispensable for phenotypic drug-susceptibility testing, but turnaround times are frequently misaligned with the tempo of respiratory deterioration in critically ill hosts. Cartridge-based nucleic acid amplification tests, including Xpert MTB/RIF Ultra and Xpert MTB/XDR, have transformed first-line care by enabling rapid confirmation of *M. tuberculosis* and selected resistance mutations directly from clinical samples. Their limitation is not speed but scope: they interrogate predefined loci, incompletely capture uncommon resistance mechanisms, and provide little information regarding competing co-pathogens (Chakravorty et al., 2017; Pillay et al., 2022).

**TABLE 3:** Tiered implementation framework for precision diagnosis and management of drug-resistant tuberculosis in resource-limited settings.

Healthcare level	Diagnostics	Therapeutic capacity	Priority
Primary health center	Clinical evaluation, Chest X-ray, Xpert MTB/RIF Ultra, HIV testing	Standard WHO-recommended DR-TB regimen initiation, referral of complicated cases	Early diagnosis and rapid treatment initiation
District/Regional hospital	Culture, phenotypic DST, Line Probe Assays, Chest CT (where available), fungal biomarkers	Individualized regimen adjustment, management of common co-infections, therapeutic drug monitoring where available	Confirmation of resistance and optimization of therapy
National Reference/Tertiary Centre	Whole-genome sequencing, mNGS, advanced imaging, multi-omics (research/specialized centers)	Precision medicine, expert multidisciplinary management, host-directed therapies within clinical trials, comprehensive therapeutic drug monitoring	Management of complex DR-TB and immunocompromised patients

Adapted from the WHO Consolidated Guidelines on Tuberculosis (Module 3: Diagnosis, 2024), the WHO Operational Handbook on Tuberculosis (Module 3: Diagnosis, 2024), and the World Health Organization [WHO], 2025 WHO Global Tuberculosis Report 2024. Recommendations should be implemented according to local laboratory capacity, healthcare infrastructure, and national tuberculosis program priorities (WHO consolidated guidelines on tuberculosis; World Health Organization [WHO], 2026 WHO operational handbook on tuberculosis: module 3: diagnosis: rapid diagnostics for tuberculosis detection, 3rd ed).

### 4.2 Whole-genome sequencing (WGS)

Whole-genome sequencing (WGS) enables resistance prediction across multiple drug classes, detection of mixed-strain infection, identification of heteroresistance, and high-resolution mapping of transmission (Gygli et al., 2019). When linked to curated mutation catalogs, concordance with phenotypic susceptibility testing is high for rifampicin, isoniazid, and fluoroquinolones, and continues to improve for newer agents. Increasingly, the bottleneck is no longer sequence generation but interpretation: converting genomic variation into clinically intelligible treatment recommendations with transparent confidence metrics. Direct-from-sample sequencing and portable platforms may further compress turnaround times, but implementation will depend on quality assurance, bioinformatics governance, and integration into routine clinical workflows rather than technology alone (Miotto et al., 2017; Allix-Béguec et al., 2018). For immunocompromised patients, unbiased pathogen detection is equally transformative. Despite its high accuracy for resistance prediction and molecular epidemiology, implementation of WGS remains limited in many high-burden settings because of infrastructure requirements, bioinformatics expertise, quality assurance, and turnaround time. Furthermore, WGS cannot reliably predict resistance for all anti-tuberculosis drugs, particularly where resistance mechanisms remain incompletely characterized. Therefore, WGS currently serves as a complementary tool rather than a replacement for phenotypic drug susceptibility testing in many clinical settings.

### 4.3 Metagenomic next-generation sequencing (mNGS)

Shotgun metagenomic next-generation sequencing (mNGS) can identify bacteria, fungi, DNA viruses, RNA viruses, and parasites from bronchoalveolar lavage, tissue, or plasma without prespecified targets. This approach is particularly valuable when standard panels are negative, when mixed infection is suspected, or when opportunistic pathogens fall outside routine diagnostic pathways (Liu et al., 2024). Across recent studies of severe pneumonia and immunocompromised hosts, mNGS has repeatedly increased pathogen yield and shortened time to microbiological clarification. Yet the major challenge is epistemic rather than technical: sequence detection does not establish causation. Clinical value depends on disciplined interpretation that incorporates organism burden, sample provenance, host immune state, radiology, inflammatory trajectory, and the prior probability of disease (Xin et al., 2025; Sun et al., 2026). Despite its considerable diagnostic potential, widespread implementation of mNGS remains challenging in many high-burden, resource-limited settings. Current barriers include the high cost of sequencing platforms and consumables, limited laboratory infrastructure, shortage of trained bioinformatics personnel, and difficulties in standardized data analysis and interpretation. Interpretation of mNGS results also requires careful clinical correlation because detection of microbial nucleic acids does not necessarily distinguish airway colonization, contamination, latent infection, and true invasive disease, particularly in immunocompromised patients with complex respiratory microbiota. Turnaround time and sustainable reimbursement mechanisms also remain important considerations for routine clinical adoption. Consequently, current WHO guidance recommends that advanced sequencing technologies should complement rather than replace established rapid molecular diagnostics, with implementation prioritized in national reference laboratories and specialized centers where they are most likely to improve patient management. As sequencing costs continue to decline and analytical workflows become more standardized, broader integration of mNGS into tuberculosis diagnostic algorithms may become increasingly feasible (Meehan et al., 2019).

### 4.4 Transcriptomics and host response signatures

A pathogen-only framework is increasingly insufficient. Host-response diagnostics seek to define the biological consequences of infection and may be especially valuable when multiple organisms are detected or cultures remain negative (Forum on Microbial Threats, 2007). Blood transcriptional signatures can distinguish active tuberculosis from latent infection, quantify inflammatory burden, and track treatment response. Single-cell transcriptomic studies have identified macrophage states marked by simultaneous inflammatory activation and impaired antimicrobial function, as well as exhausted T-cell programs associated with persistent disease. Proteomic and metabolomic platforms are generating candidate biomarkers linked to cavitation, relapse risk, and early therapeutic response (Sweeney et al., 2016; Li et al., 2019). The longer-term objective is not merely to diagnose infection, but to classify patients into actionable biological endotypes that guide intensity of investigation and therapy. Imaging is undergoing a parallel transition from descriptive morphology to computational phenotyping. High-resolution computed tomography remains substantially more informative than plain radiography for identifying cavities, bronchiectasis, angio-invasive lesions, airway-centered nodularity, and post-tuberculosis structural damage (Ooi et al., 2002). Artificial intelligence systems applied to radiography and CT can support triage, quantify disease extent, detect interval progression, and prioritize abnormal studies for expert review (Hansun et al., 2025; Friebe, 2026). Most AI-assisted diagnostic systems have been developed and validated using retrospective datasets, whereas prospective multicenter validation, regulatory approval, algorithm transparency, and integration into routine clinical workflows remain ongoing challenges. Several artificial intelligence (AI)-based chest radiography systems have been evaluated for tuberculosis screening and triage in high-burden settings. The World Health Organization recommends the use of computer-aided detection (CAD) systems as triage tools for chest X-ray interpretation in tuberculosis screening programs. Among these, CAD4TB has been widely validated in community-based screening studies and demonstrated good diagnostic performance in detecting pulmonary tuberculosis. Large multi-site evaluations and systematic reviews have further shown that AI-based tools can support radiological interpretation and improve screening efficiency, particularly in resource-limited settings, although they should be used as adjuncts to microbiological confirmation (Pande et al., 2016; Qin et al., 2021). Although these platforms can improve triage efficiency and facilitate early case detection, they are intended to complement rather than replace microbiological confirmation, particularly in immunocompromised patients and those with atypical radiological presentations.

Their greatest value is likely to emerge when imaging outputs are fused with microbiology, genomics, and host biomarkers in multimodal predictive models rather than used as stand-alone classifiers. These advances demand redesigned clinical pathways. In suspected DR-TB with co-infection risk, the optimal strategy is parallel rather than sequential: immediate molecular testing for tuberculosis and key resistance markers; simultaneous sampling for culture, fungal biomarkers, and respiratory multiplex PCR; early cross-sectional imaging when feasible; rapid escalation to WGS when resistance remains unresolved; and mNGS for severe, atypical, or diagnostically refractory disease. Therapeutic drug monitoring and multidisciplinary review should be embedded where cumulative toxicity, pharmacokinetic interactions, or organ dysfunction are likely. The future diagnostic unit is therefore not a single assay, but an adaptive decision system. The final challenge is equity. The populations at highest risk of delayed diagnosis and death are often those with the least access to sequencing, advanced imaging, bronchoscopy, or specialist interpretation. Unless supported by decentralized laboratory networks, digital connectivity, simplified sample workflows, and financing models tailored to high-burden settings, precision diagnostics may widen rather than reduce global disparities. The true measure of progress will not be technological sophistication, but whether mortality falls in the patients currently diagnosed too late. Although several transcriptomic signatures have shown promising diagnostic and prognostic performance, most require external validation across diverse geographic regions and immunocompromised populations before routine clinical implementation.

## 5 Therapeutic strategies: precision therapy in the era of immune complexity

**FIGURE 4:**
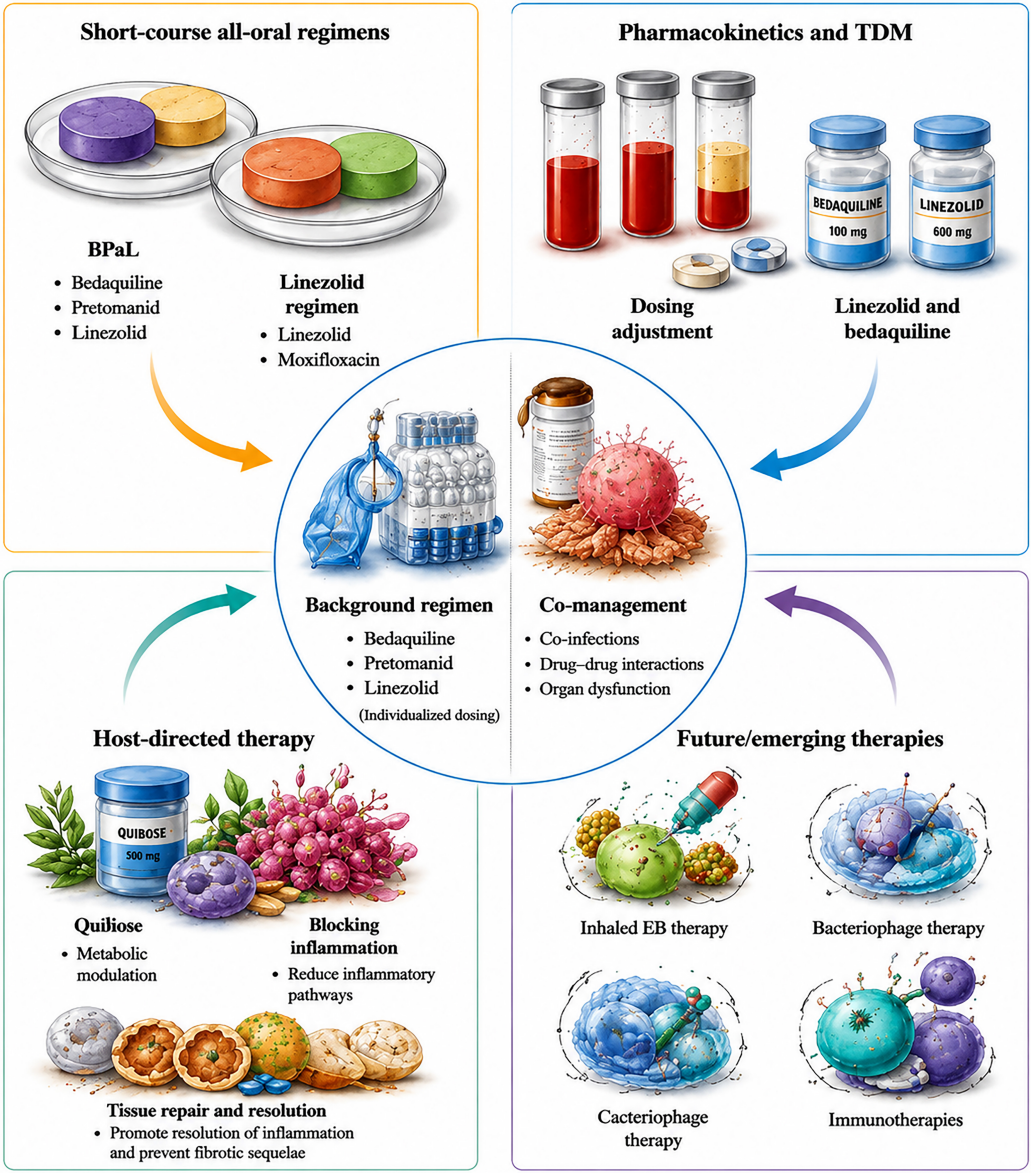
Precision treatment framework for immunocompromised patients with drug-resistant tuberculosis. Conceptual framework illustrating current and emerging therapeutic strategies for managing DR-TB in immunocompromised patients. The framework integrates optimized all-oral antimicrobial regimens, TDM, individualized dosing, management of drug–drug interactions and co-infections, host-directed therapies, and emerging interventions including inhaled drug delivery, bacteriophage therapy, and immunotherapies. The central panel highlights individualized treatment planning, while surrounding components represent complementary therapeutic strategies. Colored arrows indicate the integration of these approaches into comprehensive precision care. BPaL, bedaquiline–pretomanid–linezolid; TDM, therapeutic drug monitoring; DR-TB, drug-resistant tuberculosis. Figure created using GPT-AI and manually refined by the authors. All scientific content and therapeutic pathways were verified against the peer-reviewed literature cited in the manuscript.

Management of DR-TB with pulmonary co-infections in immunocompromised patients is undergoing a fundamental transition: from standardized regimen delivery toward adaptive, biology-informed care (Drug-resistant TB treatment, 2025). The older therapeutic model was built around prolonged, toxic, largely uniform multidrug combinations applied with limited knowledge of resistance architecture, host pharmacology, or competing pathogens. That model is increasingly obsolete. Contemporary care is now shaped by shorter all-oral regimens, rapid resistance-informed optimization, therapeutic drug monitoring, integrated treatment of bacterial and fungal co-infections, and early exploration of host-directed interventions (Figure 4 and Table 4). The central challenge is no longer simply selecting active drugs against *M. tuberculosis*, but designing regimens that remain effective within the constraints of immunosuppression, organ dysfunction, polypharmacy, and overlapping toxicities.

**TABLE 4:** Emerging therapeutics and precision approaches.

Strategy	Example	Evidence level	Advantages	Limitations	References
Short-course all-oral DR-TB regimens	BPaL (bedaquiline–pretomanid–linezolid); BPaLM (addition of moxifloxacin)	Phase II/III trials; WHO-endorsed regimens (2022–2024 updates)	High treatment success (∼85–90% in selected cohorts); reduced duration (∼6 months); avoids injectables	Limited data in severely immunocompromised patients; linezolid toxicity (myelosuppression, neuropathy)	(Conradie et al., 2020)
Individualized therapy using WGS	Whole-genome sequencing-guided regimen selection	Increasing programmatic and cohort evidence	Rapid resistance profiling; detects heteroresistance and mixed infection; supports precision prescribing	Requires infrastructure, bioinformatics expertise; variable turnaround in low-resource settings	(CRyPTIC Consortium and the 100, 000 Genomes Project et al., 2018)
Therapeutic drug monitoring (TDM)	Linezolid, bedaquiline, fluoroquinolones	Observational studies and clinical guidance	Reduces toxicity; optimizes exposure in renal/hepatic dysfunction; useful in drug–drug interactions	Limited availability; lack of standardized thresholds for all drugs	(Alsultan and Peloquin, 2014; Nahid et al., 2019)
Host-directed therapy (HDT)	Metformin, statins, NSAIDs, immune modulators	Early-phase trials and observational data	Enhances host immunity; reduces inflammation and tissue damage; potential adjunct to shorten therapy	Limited large-scale RCT evidence; unclear optimal patient selection	(Wallis and Hafner, 2015)
AI-guided treatment decision systems	Clinical decision-support tools integrating genomics + clinical data	Emerging translational studies	Integrates complex datasets; supports personalized regimens; improves decision speed	Limited validation in real-world settings; requires digital infrastructure	(Menon and Koura, 2025)
Inhaled anti-TB drug delivery	Inhaled rifampicin, clofazimine formulations (experimental)	Preclinical + early clinical studies	High local lung concentration; reduced systemic toxicity; potential for shorter therapy	Limited human data; formulation and delivery challenges	(Menon and Koura, 2025)
Antifungal–TB integrated therapy	Azole therapy with modified TB regimens (rifamycin-sparing)	Observational and pharmacokinetic studies	Enables management of TB–fungal co-infection; improved survival with early antifungal use	Major drug–drug interactions (rifampicin–azole); need for TDM	(Denning et al., 2018)
Bacteriophage therapy for bacterial co-infection	Phage therapy for MDR *Pseudomonas* and *Acinetobacter*	Case reports and early trials	Active against multidrug-resistant bacteria; personalized approach	Limited clinical evidence; regulatory and scalability challenges	(Aslam et al., 2020)

### 5.1 Optimizing anti-tuberculosis therapy and therapeutic drug monitoring

The most important advance has been the move away from injectable-based therapy toward potent all-oral regimens anchored by bedaquiline, linezolid, fluoroquinolones, pretomanid, clofazimine, cycloserine, and delamanid. WHO updates between 2022 and 2024 endorsed shorter regimens for eligible patients, reflecting accumulating evidence that treatment duration can be reduced without sacrificing efficacy when resistance is adequately characterized (Conradie et al., 2022; Riccardi et al., 2023). In selected populations with multidrug-resistant or extensively drug-resistant tuberculosis, the BPaL and BPaLM regimens (bedaquiline, pretomanid, linezolid, with or without moxifloxacin) achieved high favorable outcome rates in program and trial settings, establishing a new benchmark for regimen design (Havlir et al., 2011). Although BPaL and BPaLM regimens have demonstrated high efficacy in the Nix-TB, ZeNix, and TB-PRACTECAL trials, their generalizability to certain high-risk immunocompromised populations remains uncertain. In the ZeNix trial, people living with HIV were eligible only if they were receiving antiretroviral therapy and had a CD4 count > 100 cells/mm^3^, whereas TB-PRACTECAL included people living with HIV with a median CD4 count of 322 cells/mm^3^ (Berry et al., 2022). Consequently, prospective evidence in patients with advanced HIV infection (e.g., CD4 < 50 cells/mm^3^) is lacking. Likewise, solid-organ transplant recipients have not been specifically evaluated in prospective BPaL/BPaLM clinical trials. Therefore, the use of these regimens in these populations should be individualized, with careful attention to drug–drug interactions, toxicity, and close clinical monitoring until additional prospective evidence becomes available. In patients with advanced HIV infection (CD4 < 50 cells/mm^3^), BPaL/BPaLM may be considered when effective alternatives are limited, but treatment should ideally be undertaken in experienced centers with close monitoring for immune reconstitution inflammatory syndrome, drug toxicity, and drug–drug interactions with antiretroviral therapy (Nahid et al., 2019; Conradie et al., 2022). In solid-organ transplant recipients, careful assessment of interactions between anti-tuberculosis agents and calcineurin or mTOR inhibitors is essential, with therapeutic drug monitoring whenever feasible. Baseline and regular monitoring of complete blood count, liver and renal function, peripheral neuropathy, visual symptoms, and electrocardiography (particularly for QT prolongation) are recommended (Berry et al., 2022). Linezolid dose reduction or interruption should be considered for significant myelosuppression or neuropathy. When BPaL/BPaLM is contraindicated or intensive monitoring cannot be provided, individualized WHO-recommended all-oral regimens should be selected according to resistance profiles, comorbidities, and drug tolerability. Yet these successes should not be overgeneralized: severely immunocompromised patients, advanced liver disease, profound cytopenias, and complex co-infection states remain under-represented in pivotal studies. Precision therapy therefore begins with regimen fit, not regimen availability. In patients with renal impairment, hepatic dysfunction, malabsorption, extreme body weight, or major drug–drug interaction risk, standard dosing may be inappropriate. Therapeutic drug monitoring (TDM) is increasingly valuable for linezolid, fluoroquinolones, aminoglycosides where used, triazoles, and selected antiretrovirals. Dose individualization can preserve efficacy while limiting predictable toxicity such as linezolid-associated myelosuppression, peripheral neuropathy, optic neuropathy, QT prolongation, nephrotoxicity, or hepatotoxicity. Integration of rapid genotypic testing and whole-genome sequencing further allows early removal of ineffective agents and rational construction of high-barrier regimens (Subramanian et al., 2019; Salmanton-García et al., 2024). Current recommendations support therapeutic drug monitoring (TDM) as an adjunctive tool rather than a routine requirement for all patients receiving anti-tuberculosis therapy. TDM is particularly valuable in patients with drug-resistant tuberculosis who have delayed microbiological response, suspected malabsorption, significant drug–drug interactions, renal or hepatic dysfunction, extremes of body weight, or increased risk of toxicity, especially during treatment with linezolid, aminoglycosides, or other agents with narrow therapeutic windows. Integration of TDM with clinical assessment, microbiological response, and adverse-event monitoring can facilitate individualized dose optimization, maximize drug exposure, and reduce treatment-related toxicity in complex immunocompromised patients (Peloquin, 2002; World Health Organization [WHO], 2020)

### 5.2 Management of pulmonary co-infections and drug–drug interactions

In critically ill immunocompromised patients, delayed treatment of bacterial or fungal disease can be as lethal as undertreated DR-TB. Empirical antibacterial coverage may be justified for severe sepsis or hospital-acquired pneumonia, but should be rapidly narrowed using culture, multiplex PCR, and susceptibility data to minimize collateral selection of carbapenem-resistant organisms. Antifungal therapy requires equal precision. Chronic pulmonary aspergillosis often necessitates prolonged triazole therapy, whereas invasive mold disease may require voriconazole, isavuconazole, or liposomal amphotericin-B depending on host status and site of disease. The major barrier is pharmacology: rifampicin profoundly lowers azole exposure, while azoles can increase concentrations of calcineurin inhibitors, corticosteroids, and other co-administered agents. In many patients, treatment success depends less on drug choice than on managing interactions intelligently (Lu et al., 2024). Viral co-infections introduce a different therapeutic logic. Management of influenza or SARS-CoV-2 depends on timely antiviral therapy, oxygenation strategies, thromboprophylaxis where indicated, and prevention of secondary bacterial infection (Jheng et al., 2025). In transplant recipients and profoundly immunosuppressed hosts, cytomegalovirus requires pre-emptive or targeted therapy with structured virological monitoring. Across these scenarios, systematic review of cytochrome P450 interactions, additive QT effects, marrow suppression, and renal clearance is essential when antiviral and anti-tuberculosis agents are co-prescribed (Razonable et al., 2013).

### 5.3 Host-directed therapies and emerging precision therapeutics

**FIGURE 5:**
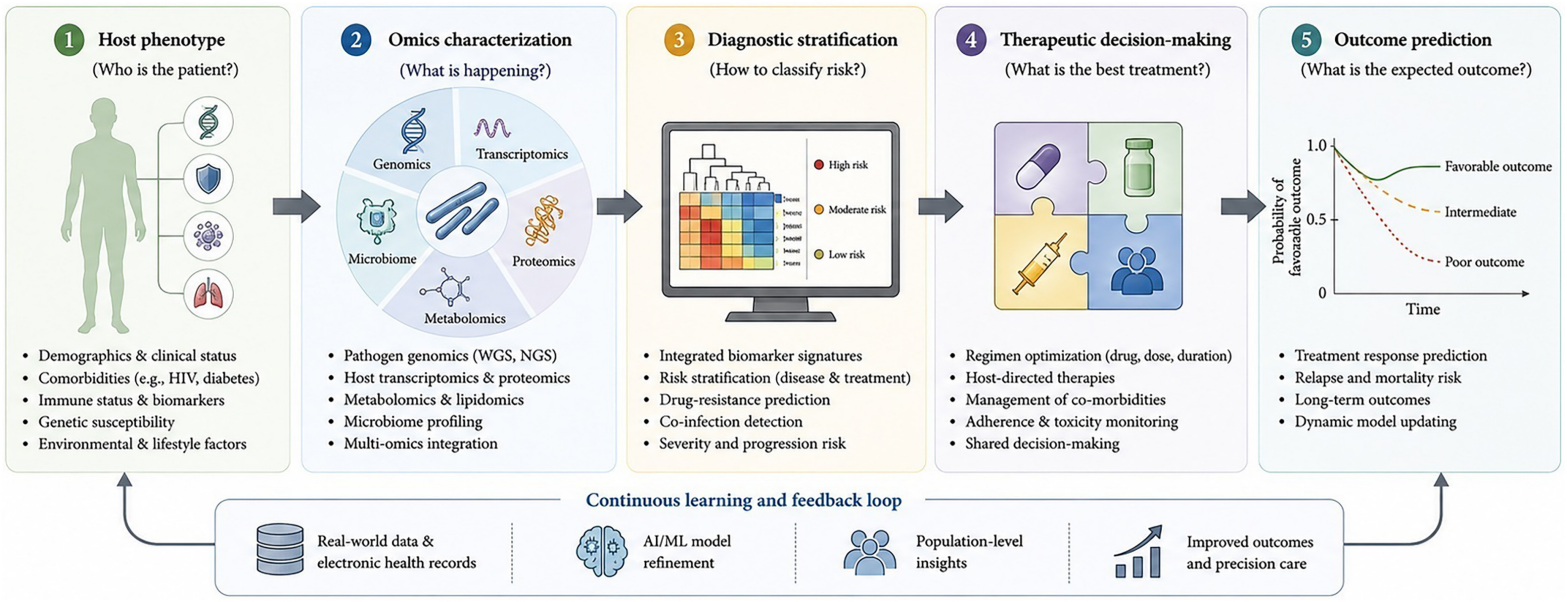
Integrated precision medicine framework for drug-resistant tuberculosis and pulmonary co-infections in immunocompromised patients. Conceptual framework summarizing a precision medicine approach to the management of DR-TB and pulmonary co-infections in immunocompromised patients. The framework integrates host phenotyping, multi-omics technologies (including genomics, transcriptomics, proteomics, metabolomics, and metagenomics), advanced molecular diagnostics, artificial intelligence-assisted clinical decision support, individualized therapeutic selection, and longitudinal outcome monitoring. Bidirectional arrows illustrate the continuous interaction between clinical data, molecular profiling, therapeutic response, and adaptive decision-making to optimize patient management. DR-TB, drug-resistant tuberculosis; AI, artificial intelligence; WGS, whole-genome sequencing; mNGS, metagenomic next-generation sequencing. Figure created using GPT-AI and manually refined by the authors. All scientific content, workflows, and clinical pathways were verified against the peer-reviewed literature cited in the manuscript.

The next frontier is host-directed therapy. Antimicrobial activity alone does not reverse immune paralysis, excessive neutrophilic tissue injury, post-inflammatory fibrosis, or persistent inflammatory endotypes that drive poor outcomes (Alipoor et al., 2026). Agents under investigation include metformin, statins (to influence lipid metabolism and inflammatory signaling), phosphodiesterase inhibitors such as CC-11050 (to reduce pathological inflammation), eicosanoid modulators, eicosanoid modulators targeting prostaglandin and leukotriene pathways, and immune checkpoint-directed approaches designed to reverse T-cell exhaustion. Corticosteroids remain established therapy for selected extrapulmonary syndromes such as tuberculous meningitis and pericarditis, but in pulmonary disease they require caution because benefit may be offset by secondary infection risk, hyperglycemia, and impaired pathogen clearance. The strategic goal of host-directed therapy is not non-specific immunostimulation, but correction of the dominant biological defect in an individual patient (Wallis and Hafner, 2015; Naicker et al., 2020). Several clinical trials are currently evaluating HDTs as adjuncts to standard anti-tuberculosis therapy. A completed Phase II trial (ClinicalTrials.gov Identifier: NCT02968927) evaluated CC-11050, everolimus, auranofin, and vitamin D3 in patients with pulmonary tuberculosis and demonstrated that CC-11050 and everolimus were safe and associated with improved recovery of lung function, although no significant improvement in sputum culture conversion was observed (The Aurum Institute NPC, 2019). Additional ongoing studies include the StAT-TB trial evaluating adjunctive pravastatin (NCT03882177) and the TB-MET-NAC trial assessing metformin with N-acetylcysteine for post-tuberculosis lung function recovery (NCT07136987). These studies are expected to provide stronger evidence regarding the role of HDTs in precision tuberculosis therapy (National Institute of Allergy and Infectious Diseases [NIAID], 2023; Open Source Pharma Foundation, 2025). Digital and computational tools are beginning to influence therapeutic delivery. Decision-support platforms integrating resistance profiles, interaction databases, laboratory monitoring, and patient-specific comorbidity data can improve prescribing safety in specialized centers. Digital adherence technologies, remote symptom capture, electronic directly observed therapy, and decentralized monitoring may reduce treatment interruption during long regimens. Emerging machine-learning models aim to predict toxicity, non-adherence, or failure before clinical deterioration becomes evident, although prospective validation remains limited Filho et al., 2025). Therapy must also be tailored to immune phenotype. In HIV, antiretroviral therapy should be initiated or optimized with careful attention to timing, immune reconstitution inflammatory syndrome, and interactions with rifamycins or newer TB drugs (Le and Shen, 2024). In transplant recipients, reduction of immunosuppression must be balanced against rejection risk. In oncology populations, coordination with chemotherapy, cellular therapy, or anti-CD20 schedules may determine both infection control and cancer outcomes (Bigotte Vieira et al., 2024). In diabetes and chronic kidney disease, optimization of glycemia, nutrition, and renal function can materially influence immune recovery and drug handling. The future of treatment is therefore not a single superior regimen, but an adaptive therapeutic ecosystem that integrates pathogen genomics, host biology, pharmacokinetics, and real-time clinical response. Long-acting formulations, inhaled delivery systems, adjunctive vaccines, bacteriophage approaches for resistant bacterial co-infection, and pragmatic platform trials in previously excluded populations are logical next steps. The decisive question is whether these advances can be translated beyond expert centers into the high-burden settings where mortality remains greatest. The concepts discussed throughout this review can be integrated into a precision medicine framework in which host phenotype, multi-omics characterization, diagnostic stratification, and individualized therapeutic decision-making collectively inform prediction of treatment response and long-term clinical outcomes (Figure 5). Such an integrated approach provides a conceptual roadmap for translating emerging molecular technologies into personalized management of drug-resistant tuberculosis in immunocompromised patients (Figure 5). The successful management of drug-resistant tuberculosis in immunocompromised patients requires a multidisciplinary approach that extends beyond antimicrobial selection alone. Optimal care is achieved through close collaboration among infectious disease physicians, pulmonologists, clinical microbiologists, pharmacists, radiologists, and specialists managing the underlying immunocompromising condition, including transplant physicians, hematologists, oncologists, and HIV clinicians where appropriate (Lange et al., 2019; Nahid et al., 2019). Such multidisciplinary teams facilitate individualized treatment selection, therapeutic drug monitoring, management of drug–drug interactions, interpretation of complex microbiological and molecular diagnostic results, and coordinated monitoring for treatment-related toxicities. This integrated model of care is increasingly recognized as an essential component of precision medicine and is particularly important for patients with complex co-infections and multiple comorbidities.

## 6 Special populations: host phenotypes that redefine risk and treatment response

Immunocompromised patients are often grouped together in clinical practice, yet this is biologically misleading. The risk, presentation, microbiological spectrum, pharmacology, and outcomes of DR-TB with pulmonary co-infection differ fundamentally according to the dominant immune defect, co-morbidity profile, and competing therapeutic priorities. A patient with advanced HIV, a kidney transplant recipient receiving tacrolimus, an older adult with frailty, and a person with poorly controlled diabetes may all meet the label of “immunocompromised”, but they do not fail host defense through the same mechanisms and should not be managed through the same pathway. Precision care therefore begins with host phenotyping rather than pathogen identification alone (Table 2).

### 6.1 People living with HIV

In people living with HIV, the defining lesion is progressive loss of CD4-positive T-cell function with secondary impairment of macrophage activation and granuloma integrity. This biology explains the higher frequency of disseminated disease, smear-negative pulmonary tuberculosis, rapid progression, and mortality (Snigdha et al., 2025). In 2023, approximately 6⋅1% of global incident tuberculosis cases occurred among people living with HIV, and the WHO African Region accounted for the majority of this burden (Sossen et al., 2025). Co-infections with recurrent bacterial pneumonia, *Pneumocystis jirovecii*, cryptococcosis, and cytomegalovirus are common at low CD4 counts. Management requires integration rather than parallelism: rapid tuberculosis treatment, carefully timed initiation or optimization of antiretroviral therapy, prophylaxis where indicated, and anticipation of immune reconstitution inflammatory syndrome (IRIS) (White et al., 2018). Rifamycin interactions with protease inhibitors and selected integrase inhibitor regimens remain clinically important, although newer all-oral DR-TB regimens may reduce some of this complexity (Naidoo et al., 2022).

### 6.2 Solid organ transplantation and hematological malignancies

In solid-organ and hematopoietic stem-cell transplant recipients, immune failure is iatrogenic and pharmacologically maintained. Calcineurin inhibitors, antimetabolites, corticosteroids, and graft-related immune dysfunction increase susceptibility to reactivation tuberculosis, invasive aspergillosis, cytomegalovirus pneumonitis, nocardiosis, and healthcare-associated Gram-negative pneumonia (Dieterich, 2007; Gold et al., 2025). Clinical presentations may be subtle despite rapidly progressive disease, and microbiological confirmation is often delayed by low organism burden or inability to tolerate invasive sampling. Treatment is dominated by pharmacokinetics: rifamycins can profoundly reduce tacrolimus, ciclosporin, and mTOR inhibitor exposure, whereas azoles may increase these concentrations to toxic levels. Successful care therefore depends on rifamycin-sparing strategies where appropriate, therapeutic drug monitoring, and continuous coordination with transplant teams (Houwen et al., 2025). In patients with solid tumors or hematological malignancy, vulnerability reflects the combined effects of malignancy itself, cytotoxic chemotherapy, targeted agents, anti-CD20 therapy, checkpoint blockade, stem-cell transplantation, and treatment-related mucosal injury. Neutropenia markedly increases the risk of bacterial sepsis and invasive mold disease, while lymphocyte dysfunction favors viral reactivation and tuberculosis progression (O’Brien et al., 2003). Pulmonary infiltrates are diagnostically challenging because infection, drug toxicity, radiation injury, hemorrhage, and tumor progression may appear radiologically similar. Therapeutic decisions often require sequencing rather than simultaneous escalation: delaying chemotherapy may compromise cancer outcomes, whereas uncontrolled infection can be immediately fatal. Overlapping marrow toxicity, particularly with linezolid or antiviral agents, further complicates regimen design (Hanania et al., 2019).

### 6.3 Diabetes, chronic kidney disease, and older adults

Diabetes mellitus has emerged as one of the most important non-communicable modifiers of tuberculosis epidemiology. Chronic hyperglycemia impairs neutrophil chemotaxis, macrophage phagocytosis, intracellular killing, and epithelial repair, and is associated with delayed sputum conversion, more extensive cavitary disease, relapse, and mortality. In parallel, diabetes increases susceptibility to chronic pulmonary aspergillosis and mucormycosis, especially in the presence of residual cavities, ketoacidosis, corticosteroid exposure, or chronic kidney disease. Management of DR-TB in diabetes is therefore inseparable from glycemic optimization, nutritional support, and review of drug absorption and renal function (Martinez and Kornfeld, 2014; Madden et al., 2025). Chronic kidney disease (CKD) and dialysis create a distinct therapeutic phenotype characterized by impaired cellular immunity, repeated healthcare exposure, vascular access devices, anemia, and altered drug clearance. These patients face increased risks of tuberculosis, bloodstream infection, and nosocomial pneumonia. Standard dosing may be inappropriate because renally cleared drugs or toxic metabolites can accumulate, while intermittent hemodialysis may unpredictably alter exposure. In this setting, therapeutic drug monitoring and close pharmacist involvement are particularly valuable (Awdishu et al., 2025). Older adults are not simply younger patients with more co-morbidity. Aging is associated with immunosenescence, reduced naïve T-cell reserves, impaired vaccine responsiveness, chronic low-grade inflammation, sarcopenia, frailty, and reduced physiological reserve. Tuberculosis may present atypically with delirium, functional decline, anorexia, or minimal respiratory symptoms, contributing to diagnostic delay. Polypharmacy increases interaction risk, while prolonged multidrug regimens are often poorly tolerated. For many older adults, the relevant clinical outcome is not microbiological cure alone, but cure achieved without catastrophic loss of function or independence (Yayan and Rasche, 2026).

### 6.4 Patients receiving biologic and targeted immunomodulatory therapy

A rapidly expanding population includes patients receiving biologic and targeted immunomodulatory therapies. Tumor necrosis factor inhibitors, interleukin antagonists, Janus kinase inhibitors, anti-CD20 agents, and other pathway-specific therapies can destabilize latent immune control of tuberculosis and increase susceptibility to opportunistic infection. Although latent tuberculosis screening before treatment initiation is now standard in many settings, breakthrough disease still occurs, particularly in high-incidence regions or during prolonged therapy. Management frequently requires temporary modification of immunosuppression, but interruption must be balanced against flare of the underlying inflammatory or malignant disease (Ji et al., 2022). Across all host groups, three principles recur. Immune phenotype should determine diagnostic breadth: the differential diagnosis in advanced HIV is not the same as that in a transplant recipient or a patient with neutropenia. Pharmacology is often as important as microbiology, because drug–drug interactions and organ dysfunction can convert an active regimen into an ineffective or unsafe one. Evidence remains inadequate, as many tuberculosis trials still exclude the patients at highest risk of death. Future studies should prioritize platform designs that deliberately enroll immunocompromised populations, incorporate co-infection endpoints, and evaluate host-adapted treatment strategies rather than assuming one regimen fits all.

## 7 Research gaps and future directions: from incremental progress to precision systems care

### 7.1 Evidence gaps

The field has progressed technologically faster than it has matured scientifically: new assays exist, new drugs exist, and new biological insights exist, yet the patients at highest risk of death are still those least represented in trials, least visible in surveillance systems, and least likely to access advanced care. The next phase of progress will depend less on isolated innovations than on redesigning research frameworks around biological heterogeneity, implementation reality, and equity. The most immediate gap is evidentiary exclusion. Many pivotal DR-TB trials underpinning shorter all-oral regimens enrolled relatively few patients with advanced HIV, severe cytopenias, solid-organ transplantation, dialysis dependence, profound malnutrition, or active fungal co-infection (Sinha et al., 2023). Yet these are precisely the populations in whom toxicity, pharmacokinetic variability, immune dysregulation, and competing pathogens most strongly determine outcome. Future trials should move beyond broad exclusion criteria and instead use stratified enrollment, adaptive platform designs, host-specific safety endpoints, and preplanned subgroup analyses that make complexity a study objective rather than a reason for omission (Conradie et al., 2020; Nyang’wa et al., 2024). Current literature often conflates colonization, co-detection, superinfection, and invasive disease, making cross-study comparison difficult and therapeutic thresholds uncertain. This problem is particularly acute for fungi, respiratory viruses, and organisms identified through metagenomic sequencing. Consensus definitions anchored to sample type, microbial burden, host phenotype, radiology, and outcome relevance are urgently needed. Multicenter prospective cohorts with serial sampling should be prioritized to define incidence, attributable mortality, and when treatment changes prognosis rather than merely treats laboratory noise (Bassetti et al., 2021). Successful implementation of emerging precision medicine strategies will also require addressing important health-system barriers in low- and middle-income countries (LMICs), where the burden of drug-resistant tuberculosis is greatest. Limited laboratory infrastructure, shortages of trained personnel, restricted access to advanced molecular diagnostics and sequencing technologies, high costs, fragmented referral systems, and inconsistent drug availability continue to constrain the adoption of precision diagnostic and therapeutic approaches. Strengthening laboratory networks, expanding workforce training, integrating new technologies within existing national tuberculosis programs, and ensuring equitable financing will be essential for sustainable implementation. Future implementation research should therefore evaluate not only diagnostic accuracy and clinical efficacy but also cost-effectiveness, scalability, and real-world feasibility across diverse healthcare settings, particularly in resource-constrained environments (Reid et al., 2019).

### 7.2 Limitations in current evidence and biomarker translation

Transcriptomic, proteomic, metabolomic, and single-cell studies have generated numerous candidate biomarkers associated with disease activity, relapse risk, treatment response, and immune dysfunction. However, most signatures remain derived from selected cohorts, measured on expensive platforms, and insufficiently validated across geography, ancestry, HIV status, diabetes, and co-infection states. The critical task is now reduction rather than discovery: converting complex signatures into robust, low-cost assays that retain performance in real-world settings. Biomarkers that cannot survive decentralization will have limited global value (Pai et al., 2016; Zhuang et al., 2024). Immunocompromised patients frequently receive combinations of anti-tuberculosis agents, azoles, antivirals, antiretrovirals, calcineurin inhibitors, chemotherapy, anticoagulants, and supportive medications. Yet prospective pharmacokinetic and drug–drug interaction data for newer tuberculosis regimens in these contexts are sparse. The field needs population pharmacokinetic modeling, exposure–toxicity analyses, genotype-informed metabolism studies, and pragmatic therapeutic drug monitoring trials that define when dose adjustment improves outcomes rather than simply changes concentrations (Thu et al., 2023). Broad immunomodulation is unlikely to benefit all patients and may harm some. The relevant question is not whether host-directed therapy works in tuberculosis, but which intervention benefits which biological endotype, at what disease stage, and with what safety trade-offs. Trials should therefore embed mechanistic biomarkers, immune phenotyping, and adaptive stopping rules, rather than treating host-directed agents as conventional add-ons to antimicrobial regimens (Wallis and Hafner, 2015). Cure is not the end of disease for many patients with DR-TB. Persistent cavities, fibrosis, bronchiectasis, pulmonary vascular dysfunction, chronic aspergillosis, exercise limitation, depression, and socioeconomic loss are common after microbiological cure. Long-term cohorts integrating lung function, imaging, recurrent infection, quality of life, and return-to-work outcomes are urgently needed. Without this shift, programs may overestimate success while undercounting disability (Gai et al., 2023). Genomics, imaging, pharmacy records, microbiology, bedside observations, and outcomes data are rarely interoperable. Precision medicine will fail if clinically relevant information remains trapped in disconnected platforms. Future infrastructure should prioritize common data models, transparent decision-support systems, auditability, and prospective validation of artificial intelligence tools for bias, safety, and generalizability before routine deployment (Memon et al., 2025). The countries bearing the greatest burden of tuberculosis and the populations bearing the greatest burden of immune vulnerability often have the least access to sequencing, bronchoscopy, CT imaging, therapeutic drug monitoring, and newer medicines. Funding strategies should therefore prioritize scalable diagnostics, workforce development, integrated TB–HIV–oncology–transplant services, and locally led research networks rather than technology transfer alone. Innovation that cannot be implemented at scale will not alter global mortality (Reid et al., 2019). The future of the field lies in moving from disease-centered care to systems care: integrating pathogen biology, host phenotype, pharmacology, recovery, and health-system context within a single adaptive framework. That transition not any single drug or test will determine whether precision medicine becomes reality for immunocompromised patients with DR-TB and pulmonary co-infections.

## 8 Conclusion

Drug-resistant tuberculosis in immunocompromised patients represents a complex clinical and biological challenge driven by dynamic interactions between host immune dysfunction, pathogen evolution, and frequent pulmonary co-infections. Recent advances in ^**^multi-omics technologies^**^, including whole-genome sequencing, metagenomics, transcriptomics, proteomics, metabolomics, single-cell analysis, and artificial intelligence–assisted diagnostics, are transforming the understanding of disease heterogeneity and enabling more precise stratification of patients. In parallel, improvements in therapeutic strategies, including individualized dosing, therapeutic drug monitoring, and emerging host-directed therapies, are reshaping treatment paradigms toward precision medicine. However, despite these advances, significant challenges remain, including limited access to advanced diagnostics, variability in biomarker validation, lack of large prospective clinical trials in immunocompromised populations, and implementation barriers in resource-limited settings. The integration of host phenotype, molecular diagnostics, and therapeutic response prediction into a unified clinical framework remains an unmet need. The conceptual framework presented in this review highlights a transition from conventional pathogen-centered management toward a ^**^comprehensive precision medicine approach^**^ that integrates host biology, pathogen genomics, and system-level diagnostics. Future research should focus on validating multi-omics biomarkers, improving real-world implementation, and ensuring equitable access to precision diagnostics and therapies across high-burden settings. Such an integrated strategy is essential to improve outcomes and move closer to global tuberculosis control and elimination goals.
